# Supplementary material for: Polymorphism in IFNλ Can Impact the Immune/Inflammatory Response to COVID-19 Vaccination in Older CMV-Seropositive Adults
Source: Vaccines (Basel). 2025 Jul 24;13(8):785. doi: 10.3390/vaccines13080785 (PMC12390593; doi:10.3390/vaccines13080785)
Supplement: Supplementary file 1 [file vaccines-13-00785-s001.zip › vaccines-3737336-supplementary.pdf]

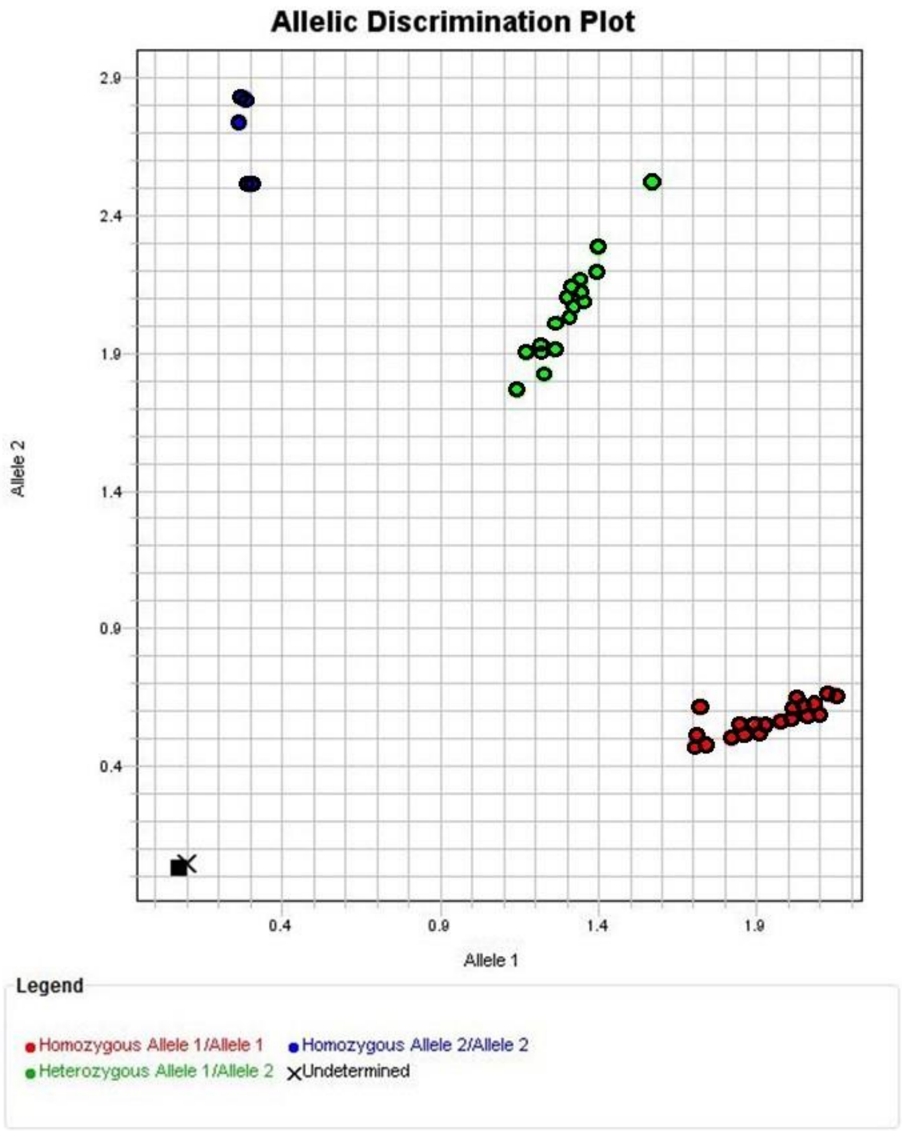

Figure S1. Representative graphic of allelic discrimination obtained after the genotyping assay for polymorphism in the il28b gene (rs12979860) in the older adults who participated in the study. In red color are presented the volunteers homozygous for allele 1, which corresponds to the nitrogenous base cytosine. In blue are presented the volunteers homozygous for allele 2, corresponding to the nitrogenous base thymine. In green are presented the heterozygous volunteers.

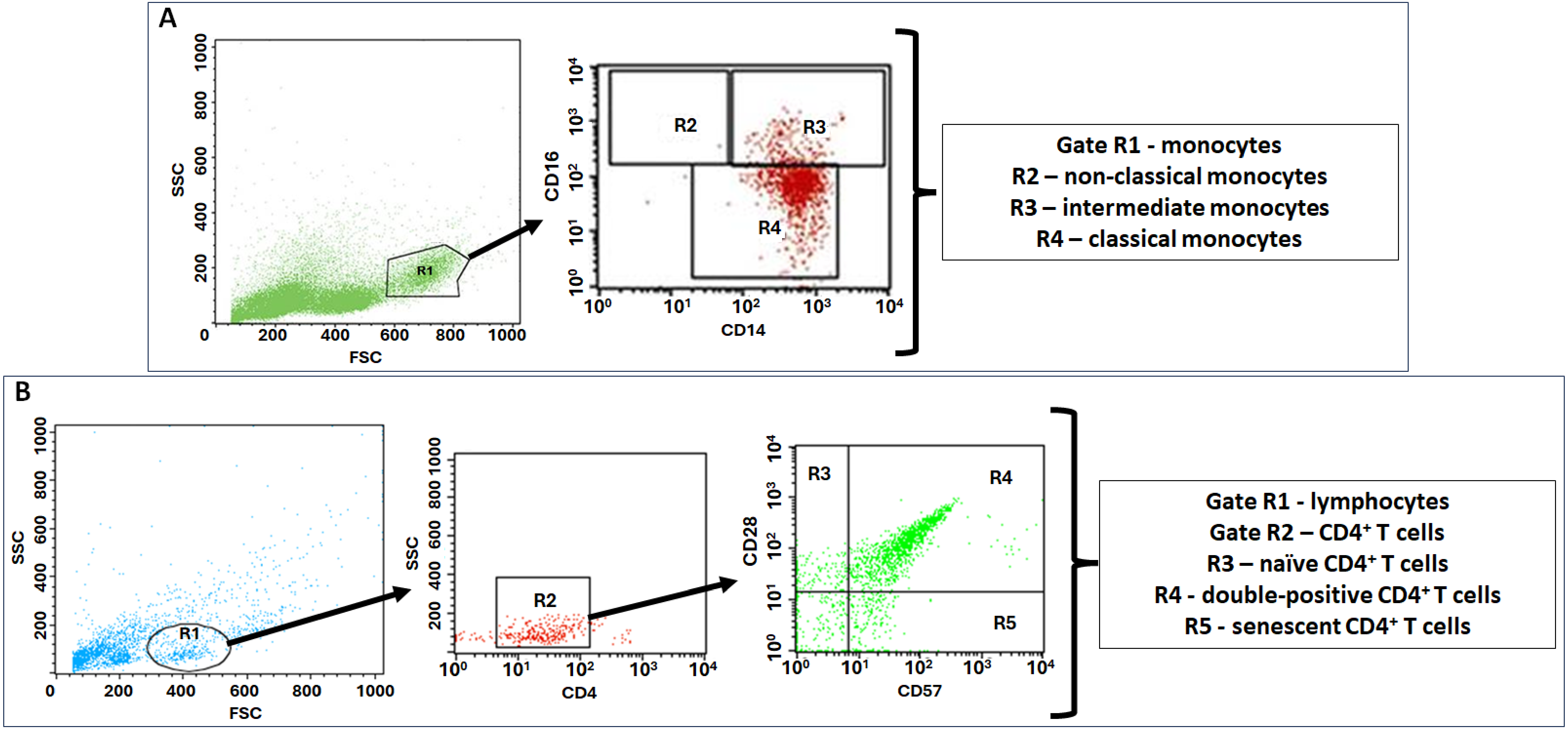

Figure S2. Representative dot plots of the flow cytometry analysis. In panel A is shown the detection of the monocyte subtypes: non-classical - CD14+CD16++; intermediate - CD14++CD16+, and classical - CD14++CD16-. In panel B is shown the detection of CD4+ T cells associated with the profile naïve (CD28+CD57−), double-positive (CD28+CD57+), and senescent (CD28-CD57-). PBMCs were stained with Abs recognizing CD16 and CD14 for monocytes evaluation or CD4, CD8, CD28, and CD57 for T cells evaluation, and were analyzed by flow cytometry, with the gating as indicated.

VNT-test (inhibition titers)														
SARS-CoV-2 strain	Wuhan		Delta variant		SARS-CoV-2 strain	Wuhan		Delta variant		SARS-CoV-2 strain	Wuhan		Delta variant	
Time points	Pre	Post	Pre	Post	Time points	Pre	Post	Pre	Post	Time points	Pre	Post	Pre	Post
Allele-1 group	Non-responders				Allele-2 group	Non-responders				Alleles-1/2 group	Non-responders			
	<20	<20	<20	<20		<20	<20	<20	<20		<20	<20	<20	<20
	<20	<20	<20	<20		<20	<20	<20	<20		<20	<20	<20	<20
	<20	<20	<20	<20		<20	<20	<20	<20		<20	<20	<20	<20
	<20	<20	<20	<20		Responders					<20	<20	<20	<20
	<20	<20	<20	<20		40	80	20	80		<20	<20	<20	<20
	<20	<20	<20	<20		<20	80	<20	<20		<20	<20	<20	<20
	<20	<20	<20	<20							<20	<20	<20	<20
	<20	<20	<20	<20							<20	20	<20	<20
	<20	20	<20	<20							<20	20	<20	<20
	<20	20	<20	20							<20	20	<20	<20
	<20	20	<20	20							<20	20	<20	<20
	<20	20	<20	<20							<20	20	<20	<20
	<20	20	<20	<20							Responders			
	Responders										<20	40	<20	<20
	<20	80	<20	20							<20	80	<20	20
	<20	80	<20	<20							<20	80	<20	<20
	<20	40	<20	<20							<20	80	<20	20
	<20	80	<20	<20							<20	160	<20	<20
	<20	320	<20	20										
	<20	40	<20	40										
	<20	40	<20	20										

Supplementary Excel File. Results of the VNT test. In black are shown the non-significant results. In red are shown the significant results (increase, at least, 2-fold) found in the titers post-vaccination time point.
